# Oral edible plant vaccine containing hypoallergen of American cockroach major allergen Per a 2 prevents roach-allergic asthma in a murine model

**DOI:** 10.1371/journal.pone.0201281

**Published:** 2018-07-30

**Authors:** Mey-Fann Lee, Chu-Hui Chiang, Ying-Lan Li, Nancy M. Wang, Pei-Pong Song, Shyh-Jye Lin, Yi-Hsing Chen

**Affiliations:** 1 Department of Medical Research, Taichung Veterans General Hospital, Taichung, Taiwan; 2 Department of Plant Medicine, National Pingtung University of Science and Technology, Pingtung, Taiwan; 3 Institute of Biotechnology, National Changhua University of Education, Changhua, Taiwan; 4 School of Medical Laboratory and Biotechnology, Chung Shan Medical University, Taichung, Taiwan; 5 Division of Allergy, Immunology and Rheumatology, Taichung Veterans General Hospital, Taichung, Taiwan; 6 Faculty of Medicine, National Yang-Ming University, Taipei, Taiwan; Chang Gung University, TAIWAN

## Abstract

**Background:**

American cockroaches (*Periplaneta americana*) are an important indoor allergen source and a major risk factor for exacerbations and poor control of asthma. We previously reported that allergen components from American cockroaches exhibit varying levels of pathogenicity. Sensitization to major American cockroach allergen, Per a 2, correlated with more severe clinical phenotypes among patients with allergic airway diseases.

**Materials and methods:**

In this study, we examined whether oral plant vaccine-encoding full-length Per a 2 clone-996 or its hypoallergenic clone-372 could exert a prophylactic role in Per a 2-sensitized mice. The cDNAs coding Per a 2–996 and Per a 2–372 were inserted into TuMV vector and expressed in Chinese cabbage. Adult female BALB/c mice were fed with the cabbage extracts for 21 days and subsequently underwent two-step sensitization with recombinant Per a 2.

**Results:**

Per a 2-specific IgE measured by in-house ELISA in the sera of Per a 2-372-treated groups were significantly lower than in the control groups after allergen challenge but not the Per a 2-996-treated group. Moreover, Per a 2–372 vaccine markedly decreased airway hyper-responsiveness and infiltration of inflammatory cells into the lungs, as well as reduced mRNA expression of IL-4 and IL-13 in comparison with the control mice.

**Conclusion:**

Our data suggest that oral administration of edible plant vaccine encoding Per a 2 hypo-allergen may be used as a prophylactic strategy against the development of cockroach allergy.

## Introduction

Cockroaches are an important indoor allergen source and a major risk factor for development and exacerbations of asthma. German cockroaches are dominant in temperate regions, whereas American cockroaches (*Periplaneta americana*) are prevalent in warm and humid tropical and subtropical regions [1–4]. The prevalence of American cockroach allergy as determined by skin test ranges from 40~57% in Taiwan [1, 5], 44~60% in Thailand [6], 55~79% in Brazil [7], and 53% in India [8]. Previous studies have characterized 12 groups of allergenic components, namely Per a 1~12, from *P*. *americana* [9–12]. We previously reported that allergens from American cockroaches exhibit varying levels of pathogenicity. Sensitization to Per a 2 correlated with more severe clinical phenotype and inflammation among patients with airway allergy [11]. Furthermore, we found Per a 2 protein existed abundantly in roach feces and was more resistant to decomposition up to one year in the environment, [12] which would tend to make it more likely to persist in the environment.

It has been reported that a reduction in cockroach allergens below clinical relevant thresholds might be beneficial [13–15]. However, complete eradication of cockroach infestations in workplaces and homes is extremely challenging. In addition to traditional antihistamines and glucocorticoids with various delivery systems, a number of novel biologic target agents for the treatment of allergic inflammation have emerged. However, allergen-specific immunotherapy remains the only approach to establish specific immune tolerance and potentially cure the disease [16, 17]. Traditional immunotherapy for cockroach allergy is based on crude extracts containing a wide variety of undesirable proteins and may involve a long and cumbersome treatment course, lasting up to 5 years or more [17, 18].

Plant systems capable of producing vaccine antigens either by transgenic approach or a plant viral vector have the advantages of low cost, easily scalable production, and safety. Over the past two decades, several studies have proven the efficacy of plant-made vaccines delivered orally against infectious diseases in animal models and in human clinical trials [19–25]. This approach has also been extended to the field of allergen expression. The feasibility of allergen-specific immunotherapy using plant-derived vaccines has also been demonstrated recently [26–28]. However, cockroach immunotherapy with plant-expressed allergen vaccine has yet to be studied. TuMV belongs to the genus *Potyvirus*, one of the most important groups of positive sense ssRNA plant viruses infecting a vast array of crops worldwide. Several infectious clones of TuMV have been generated as useful molecular tools for studying the viral expression of heterologous open reading frames in plants [29, 30].

We previously identified three linear IgE-binding epitopes on Per a 2 [31]. On this basis, we generated a recombinant hypoallergenic clone 372 of Per a 2, which containing only one IgE- binding epitope of Per a 2 sequence 200–211 [31], as the candidate for a low-risk immunotherapy vaccine. In this study, we examined whether edible plant vaccine encoding Per a 2 full-length clone 996 or its hypoallergenic clone 372 could exert a prophylactic effect in roach-allergic asthmatic mice.

## Materials and methods

### Construction of the pTuMV-Per a 2–996 and -372 recombinant plant viruses

The infectious viral vector, p35S-TuMV CPGFP, was a gift from Dr. Yeh SD (the Biotechnology Center of National Chung Hsing University) and the construction has been described elsewhere [29]. The gene fragments of mature Per a 2–996 and partial clone 372 were amplified from a previous construct pET30-Per a 2 [11]. The primers, 5’Per a 2Nco (5’CAACCATGGTGGATCCAGTCGTCGTTCCTCTG 3’) and 3’Per a 2Nhe (5’GATGCTAGCCAGTTCTTCTACGGATTTTG 3’) were used to introduce *Nco*I and *Nhe*I sites (underlined) to Per a 2 at nt 61 and 1056 position, respectively. The primers, 5’ Per a 2-372Nco (5’CAACCATGGTGCCTGTATCAAATAACGTGG 3’) and 3’ Per a 2-372Nhe (5’GATGCTAGCTACGAGAGGTACGTAAGTGAAG 3’) were annealed at positions nt 262 and 1056 of Per a 2, respectively. The amplified fragments were cloned to *Nco*I/*Nhe*I-digested p35S-TuMVCPGFP to create the recombinant clones pTuMV-Per a 2–996 and pTuMV-Per a 2–372.

### Preparation of plant extracts and quantification of recombinant proteins

Recombinant virus cultures were initially obtained by inoculation of purified plasmid DNA into the local lesion host *Chenopodium quinoa* Wild. Subsequently, the individual local lesions were mechanically transferred to systemic host *Brassica campestris* L. var. *chinensis*. Leaves of the Chinese cabbage infected with TuMV alone or TuMV-Per a 2–996 or 372 were harvested at 20 to 40 days postinoculation (dpi) and homogenized in a blender with an equal volume of PBS. The homogenate was centrifuged at 5000 g for 10 min, filtered through gauze and lyophilized. Protein concentrations were determined by the Bradford method according to the Bio-Rad microassay procedure. Recombinant protein quantification of TuMV-Per a 2–996 and 372 were performed by densitometric analysis using image analysis software (Kodak, Rochester, NY, USA) to calculate band intensities and comparing to *E*.*coli*-derived rPer a 2 and rPer a 2–372 as the standard, respectively.

### Animals

Six-week-old female BALB/c mice were purchased from the National Laboratory Animal Center, Taiwan, and raised under specific pathogen-free conditions. All animal experiments were reviewed and approved by the Institutional Animal Care and Use Committee of Taichung Veterans General Hospital.

### Experimental design

**Fig 1:**
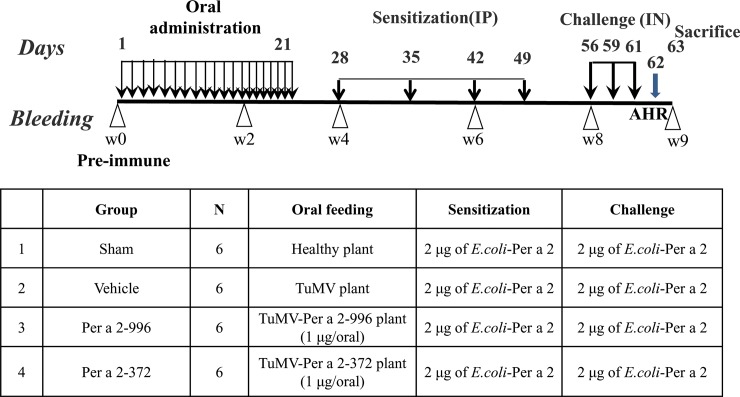
Prophylactic scheme of Per a 2-transfected plant oral feeding on rPer a 2-sensitized mice. To assess the effect of prophylactic plant vaccine, mice received a total of 21 days intragastrical administration of plant extracts containing 1 μg of Per a 2–996 or -372, or controls. One week after the final feeding, mice received intraperitoneal (IP) sensitization four times with 2 μg of *E*.*coli*-expressed recombinant Per a 2 weekly. One week after the final sensitization, mice received intranasal (IN) challenge with rPer a 2 on three consecutive days. Then, airway hyperresponsiveness (AHR) was performed on day 62 and all mice were sacrificed on day 63.

The experimental protocols of murine model are described in **Fig 1**. Six-week-old female BALB/c mice were divided into four groups with six mice each, as follows: sham group (oral healthy plant extract), vehicle group (oral TuMV viral plant extract), Per a 2–996 group (oral full-length Per a 2-viral plant extract), and Per a 2–372 group (oral partial Per a 2-viral plant extract). BALB/c mice were intragastrically (IG) administered 500 μl of plant extracts in PBS containing 1 μg of Per a 2–996 or -372 once a day for 3 weeks before sensitization. Sham and vehicle groups were fed with equal amount of plant extracts from healthy or TuMV viral plant. One week following oral feeding, all mice were then injected intraperitoneally (IP) with 2 μg of rPer a 2 four times in a volume of 200 μl on days 28, 35, 42, and 49. On days 56, 59, and 61, all mice were intranasally (IN) challenged with the same sensitized allergen. Serum samples were collected from the retro-orbital venous plexus bi-weekly and stored at -20°C until analysis. To check the response of sensitization, airway hyperresponsiveness (AHR) was assessed on day 62. All mice were sacrificed on day 63 and spleen, blood, bronchoalveolar lavage fluid (BALF), and lung were collected for further study.

### Measurement of specific antibodies by ELISA

Serum-specific IgE, IgG1 and IgG2a were determined by ELISA with the required antibodies purchased from BD Pharmingen (San Jose, CA, USA). Microtiter plates were coated with specific antigens for 2 hours at 37°C. After washing with PBST, plates were blocked with 2% BSA for 2 hours at room temperature. Sera were diluted (1:10 for IgE or 1:100 for IgGs) in PBST and incubated at room temperature for 2 hours. For IgE measurement, the plates were incubated with biotin-conjugated rat anti-mouse IgE (1:1000) for 2 hours at room temperature. Subsequently, horseradish peroxidase-conjugated streptavidin (1:4,000) (Sigma, St Louis, MI, USA) was added for 1 hour and developed by adding 2,2’-azino-bis(3-ethylbenzthiazoline-sulfonic acid (ABTS, Sigma) as substrate for 30 minutes. For IgG measurement, the plates were incubated with horseradish peroxidase-conjugated rabbit anti-mouse IgG1 or IgG2a (1:1000) for 2 hours at room temperature and developed by adding ABTS as above. Then, the optical density was analyzed on a Sunrise Absorbance Reader (TECAN, Austria) at 415 nm.

### Measurement of airway hyperresponsiveness (AHR)

The airway resistance of mice was measured by whole-body Buxco mouse plethysmography (Buxco, Troy, NY, USA) 24 hours following challenge. Mice were placed in the main chamber and challenged with aerosolized methacholine at concentrations of 6.25–50 mg/ml generated by a nebulizer (Buxco aerosol distribution system). The degree of bronchoconstriction was measured and averaged for 3 minutes after each nebulization. Airway responsiveness was expressed as enhanced pause (Penh) by the following equation: Penh = pause × (PEP/PIP). Pause, PEP and PIP representing expiration time, peak expiratory pressure, and peak respiratory pressure, respectively.

### Lung histopathology

At the end of the experiments, the lungs were removed and processed for routine histologic analysis. Briefly, the lung tissue was fixed with 10% formalin and embedded in paraffin. Four-micrometer sections were cut and stained with hematoxylin and eosin (H&E) and Periodic Acid-Schiff (PAS). Inflammatory infiltrates were examined by light microscopy and corresponding images were shot using an Olympus BX51 microscopic/DP71 Digital Camera System (Nagano, Japan).

### Measurement of cytokine production

Splenocytes were cultured in 24-well flat-bottomed plates at a concentration of 1×10^6^ cells/ml and stimulated with specific allergens for each group at 37°C for 2–5 days. The culture supernatants were collected at each time interval and stored at -20°C until use in the cytokine assay. The levels of IL-13, IL-10, and interferon (IFN)-γ in the culture supernatants were measured with murine ELISA development kits (PeproTech, Rocky Hill, NJ), according to the manufacturer’s instructions.

### Real-time PCR

To get a better idea of how various molecules are expressed in splenocytes from sensitized mice, we further used quantitative PCR on a StepOnePlus^TM^ system (Applied Biosystems, CA, USA) to measure the expression of cytokines. Predesigned primer sequences are listed in **Table 1**. In brief, cDNA were prepared from 1 μg total RNA using a SuperScript III kit (Invitrogen, Carlsbad, CA, USA). A total volume of 10 μl of PCR mixture, which included 5 μl of Real-Time SYBR Green/ROX PCR master mix from Applied Biosystems (Life Technologies, Carlsbad, CA, USA), 4 μl of double-distilled H_2_O, and 1 μl of template cDNA, were added in each well of the PCR array. PCR amplification was conducted with an initial 10-min step at 95°C followed by 40 cycles of 95°C for 15 sec and 60°C for 1 min. The fluorescent signal from SYBR Green was detected immediately after the extension step of each cycle, and the cycle at which the product was first detectable was recorded as the cycle threshold. Data were imported into an Excel database and expressed as fold change between medium alone and antigen-stimulated cells after correction by the housekeeping gene β-actin.

**Table 1:** The sequences of gene-specific primers used in real-time PCR.

Gene (mouse)		Sequences	Produced size (bp)
**IL-4**	F	5’ AGC CAT ATC CAC GGA TGC GAC AAA 3’	176
	R	5’ AAT ATG CGA AGC ACC TTG GAA GCC 3’	
**IL-13**	F	5’ AGA CCA GAC TCC CCT GTG CA 3’	123
	R	5’ TGG GTC CTG TAG ATG GCA TTG 3’	
**IL-10**	F	5’ CCA AGC CTT ATC GGA AAT GA 3’	155
	R	5’ AGG GGA GAA ATC GAT GAC AG 3’	
**IFN-γ**	F	5’ GGC CAT CAG CAA CAA CAT AAG CGT 3’	118
	R	5’ TGG GTT GTT GAC CTC AAA CTT GGC 3’	
**IL-6**	F	5’ CCA TCC AGT TGC CTT CTT G 3’	223
	R	5’ AAG TGC ATC ATC GTT GTT CAT AC 3’	
**TNF-α**	F	5’ CAT CTT CTC AAA ATT CGA GTG ACA A 3’	175
	R	5’ TGG GAG TAG ACA AGG TAC AAC CC 3’	
**β-actin**	F	5’ GGCCAACCGTGAAAAGATGA 3’	251
	R	5’ CACGCTCGGTCAGGATCTTC 3’	

### Statistical analysis

Statistical analysis was performed with SPSS version 12.0 software (SPSS Inc., Chicago, IL, USA) using appropriate methods. *P*-values less than 0.05 were considered significant.

## Results

### Expression and quantification of recombinant Per a 2–996 and Per a 2–372 by the TuMV vector

**Fig 2:**
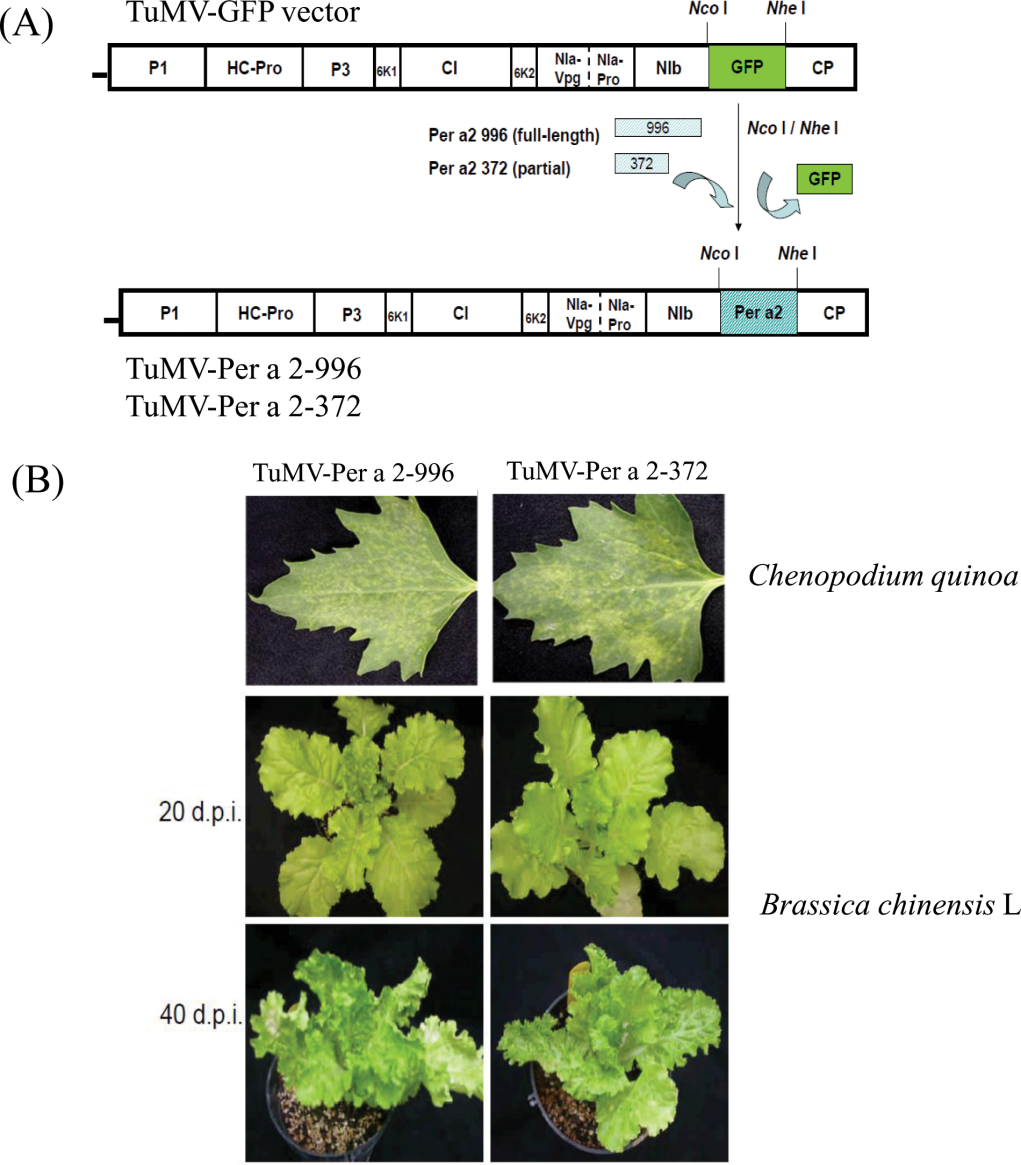
The construction of the Per a 2 gene in Turnip mosaic virus (TuMV) vector. (A) Two different sizes of DNA fragments, one containing the full-length of Per a 2 gene, named 996, and the other containing the central region of Per a 2 gene, named 372, were replaced into the GFP gene in TuMV vector. (B) Plasmids of TuMV-Per a 2–996 and 372 were inoculated into C. quinoa. Symptoms were observed 7–8 days post-inoculation (dpi). Then the local lesion on C. quinoa were used as a viral source to inoculate into Brassica chinensis L. Plants inoculated by TuMV-Per a 2–996 and TuMV-Per a 2–372 showed obvious symptoms at 40 dpi.

A schematic diagram of constructs of pTuMV-Per a 2–996 and pTuMV-Per a 2–372 is shown in Fig 2A. Symptoms developed in the local lesion host of *Chenopodium quinoa* and *systemic host Brassica chinensis* L. are shown in Fig 2B.

**Fig 3:**
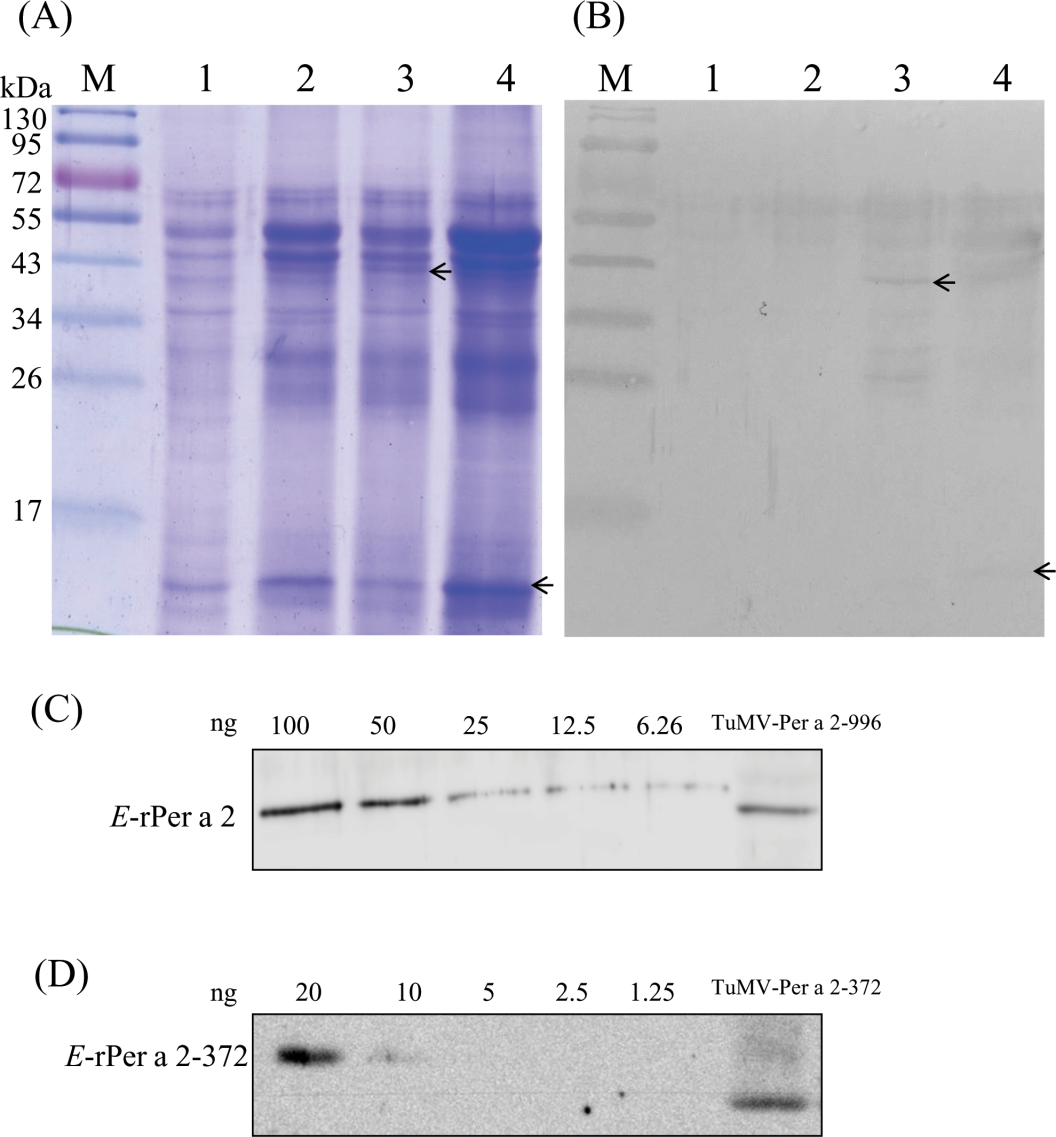
Expression analysis of American cockroach allergen Per a 2 in TuMV recombinants-infected Chinese cabbage. (A) Coomassie blue-stained SDS-PAGE and (B) immunoblotting of plant extracts from each experimental group with rabbit anti-rPer a 2-specific antibody. Lane 1, protein extract from wild-type plant (sham group); lane 2, extract from control plant infected with the viral vector alone (vehicle group); lane 3, extract from plants infected with TuMV-Per a 2–996; lane 4, TuMV-Per a 2–372 group; lane M, Pre-stained markers. (C) Recombinant protein quantification of TuMV-Per a 2–996 and 372 (D) were performed by densitometric analysis to calculate band intensities and comparing to *E*.*coli*-derived rPer a 2 and rPer a 2–372 as the standards, respectively.

The cDNAs coding for full-length Per a 2 (996) and its partial clone (372) were inserted into TuMV vector and expressed in cabbage. A band around the predicted size was visualized and confirmed by immunoblotting in plant extracts infected with TuMV-996 and 372, respectively (**Fig 3A and 3B**). The yields of viral-expressed rPer a 2–996 and 372 in plant extracts were measured by immunoblotting using *E*.*coli*-derived rPer a 2 and rPer a 2–372 as the standards (**Fig 3C and 3D**). There was a statistically significant correlation between recombinant proteins and intensity levels of the standard bands (data not shown). The levels of TuMV-expressed Per a 2–996 and 372 were 2.4 and 1.21 mg per gram of extracted plant total protein, respectively.

### The effects of edible plant vaccine on preventing the development of allergen-specific IgE antibodies

**Fig 4:**
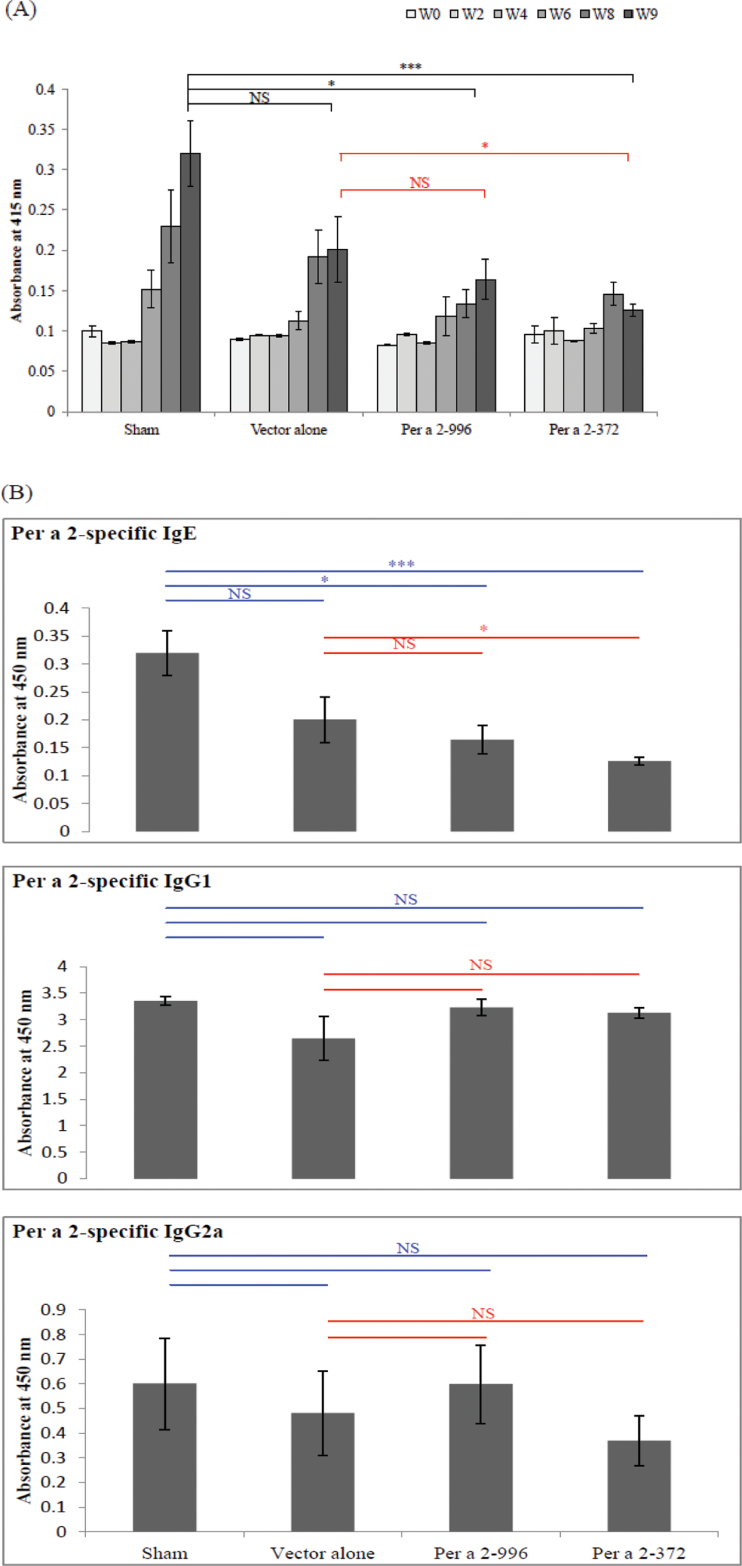
Specific antibody levels in Per a 2 sensitized mice. (A) Per a 2-specific IgE antibodies in the sera at the indicated weeks. (B) Serum levels of Per a 2-specific IgE, IgG 1 and IgG2a among groups at week 9 determined by ELISA. Results are mean±SEM of 5–6 mice from each group. *p<0.05; ***p<0.001 by one-way analysis of variance with the Bonferroni multiple range test.

To investigate whether oral administration of plant-expressed cockroach allergens could prevent the development of experimental allergy, adult female BALB/c mice were fed with plant extracts for 21 days and were then sensitized to cockroach allergen. First, we examined the systemic effects of prophylactic plant vaccine on levels of allergen-specific IgE antibody in the blood from four groups of mice. Serum Per a 2-specific IgE levels were time-dependently elevated in the sham group in weeks 4 to 9, indicating that these mice had been sensitized to American cockroach allergen Per a 2. However, the levels of specific IgE antibodies in mice of Per a 2-996- and Per a 2-372-treated groups were significantly lower than those in the sham group on week 9 (**Fig 4**A). However, there were no significant differences in the levels of IgG1 and IgG2a among any of the four groups (**Fig 4B**). This result suggests that oral administration of cockroach allergen-encoding plant vaccine prevented the development of cockroach allergen-specific IgE in mice exposed to cockroach allergens later in life.

### The effects of edible plant vaccine on airway hyperresponsiveness (AHR)

**Fig 5:**
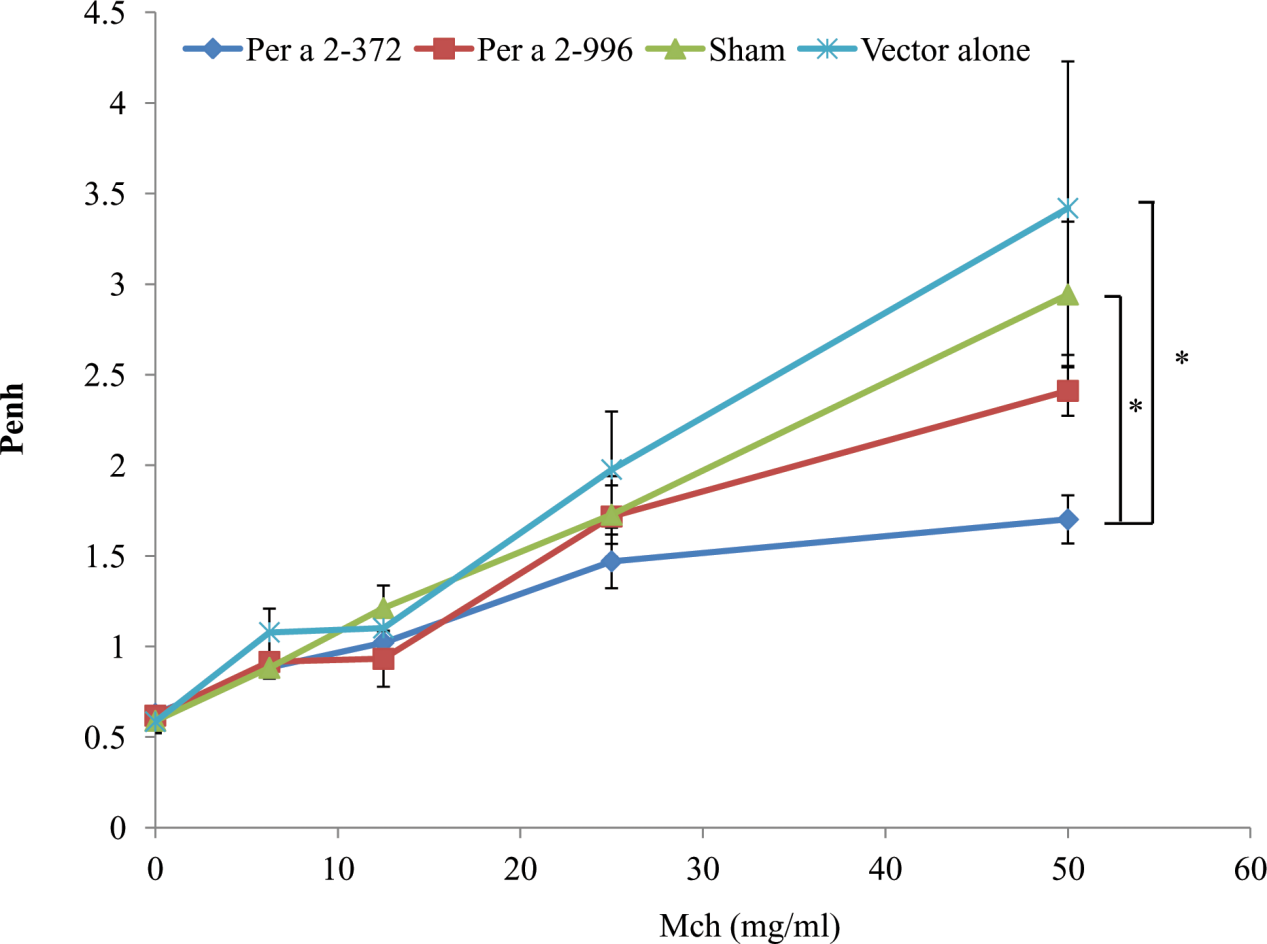
Effect of plant vaccine on airway hyperresponsiveness in rPer a 2-challenged mice. Mean enhanced pause (Penh) values were evaluated at indicated doses of methacholine (Mch) in the four groups (n = 5–6 per group). *p<0.05 by one-way analysis of variance with the Bonferroni multiple range test.

To examine the prophylactic potential of edible plant vaccine on the development of allergic asthma, mice underwent IP sensitization and subsequent IN challenge with rPer a 2. One day after the final challenge, mice were treated with saline aerosol followed by increasing concentrations of methacholine aerosol, and Penh was calculated. In the sham and vector alone groups, mice showed increased Penh upon methacholine exposure. In the allergen-fed groups, though levels of Per a 2-specific IgE decreased in both groups in compared with the sham group as shown in Fig 4, only the Per a 2-372-treated group showed a significant reduction of Penh (deep blue line) but not the Per a 2–996 treated group (red line), as shown on Fig 5. This result suggests that oral administration of hypoallergen-encoding plant vaccine attenuated airway hyperresponsiveness in mice.

### The effects of edible plant vaccine on expression of cytokine genes in immune cells

**Fig 6:**
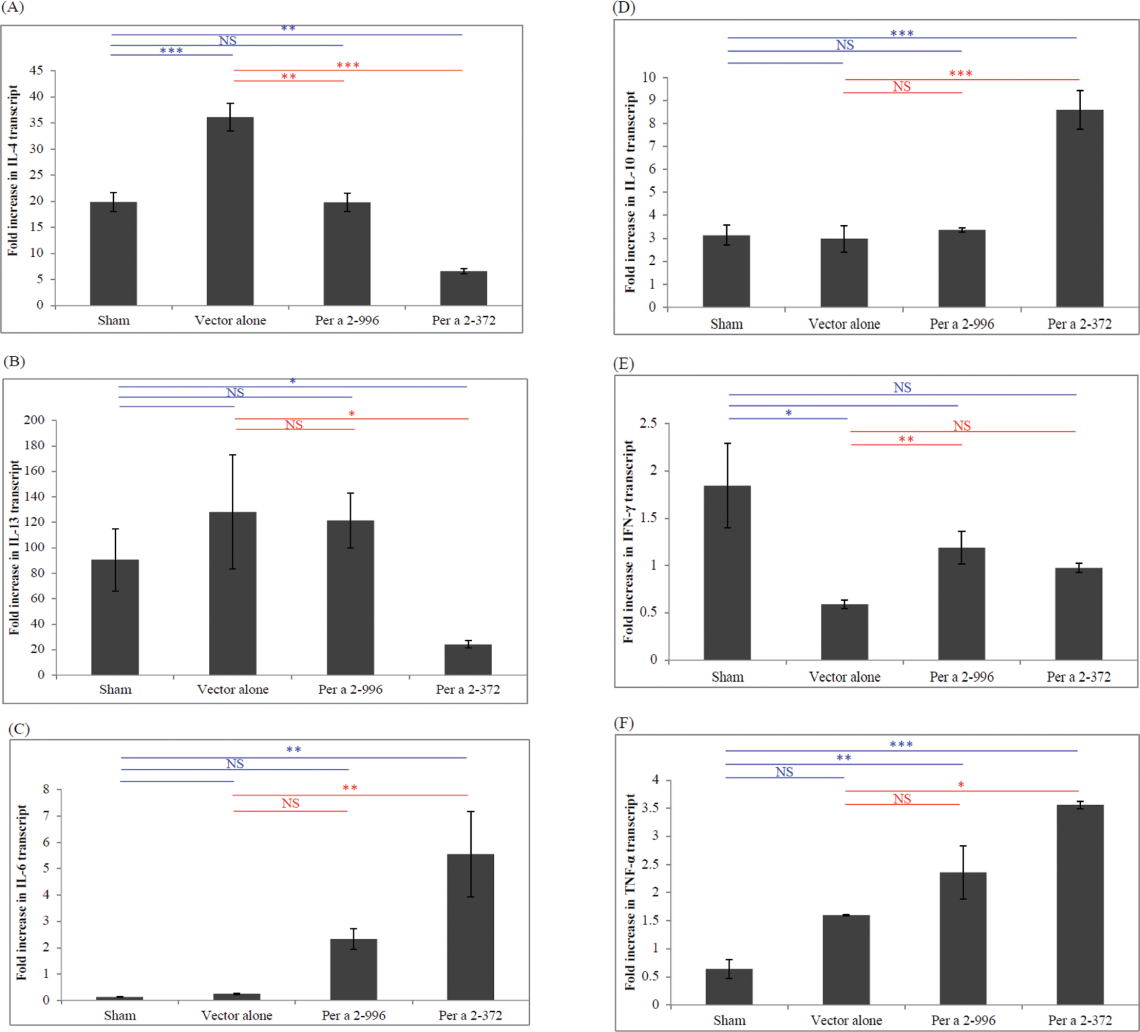
Cytokine mRNA levels of splenocytes from each group of mice by real-time PCR. (A) IL-4, (B) IL-13, (C) IL-6, (D) IL-10, (E) Interferon γ (IFN-γ), and (F) TNF-α. Data are expressed as mean of increasing fold ±SEM (n = 5–6 per group). The statistical significance of differences among groups was assessed by the Dunnett’s test. * denoted *p*<0.05; **p<0.01; ***p<0.001.

To examine the effects of plant vaccine on mouse immune cells, splenocytes from each group were incubated with rPer a 2 for 3 days. The cytokine mRNA expression in response to rPer a 2 were determined by real-time PCR as shown in **Fig 6**. There was a marked down-regulation of mRNA expression of IL-13 and IL-4 in the Per a 2–372 group compared with that of either the sham group or vector alone group (**Fig 6A and 6B**). Interestingly, mRNA levels of IL-10, IL-6, and TNF-αwere significantly elevated in the Per a 2–372 group compared with those of the sham group or vector alone group (**Fig 6C, 6D and 6F**). Moreover, the IFN-γ mRNA level was decreased in the virus-infected (vector alone) groups compared with the sham group (**Fig 6E)**. Thus, it appears that oral administration of hypoallergen-encoding plant vaccine modulated cytokine gene expression in splenocytes, contributing to the inhibition of allergic inflammation in rPer a 2-sensitized mice.

The protein levels of IL-13, IFN-gamma and IL-10 were shown at supplement S1A–S1C Fig.

### The effects of edible plant vaccine on airway inflammation in lung tissue

**Fig 7:**
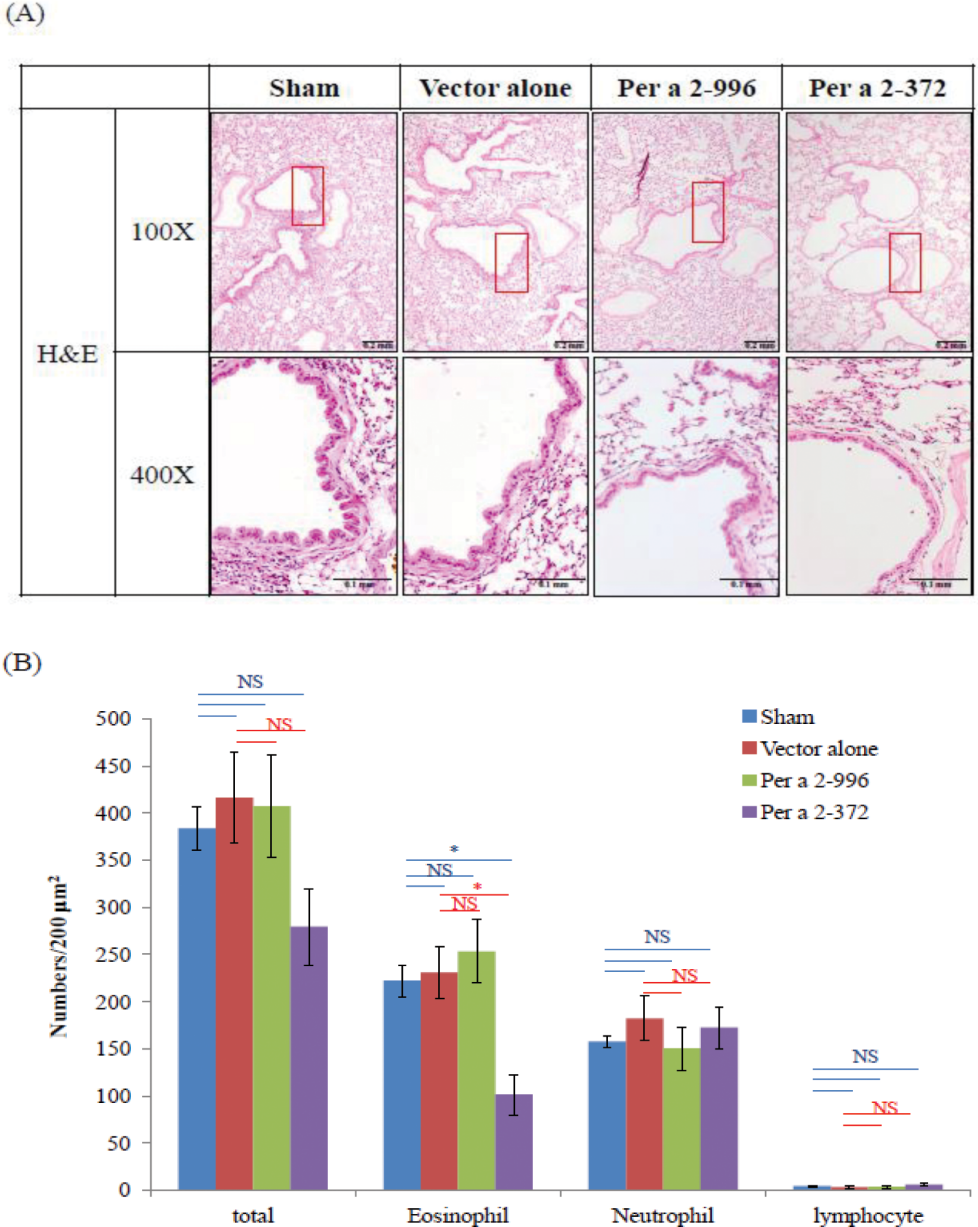
Effect of plant vaccine on histopathology of lung inflammation. (A) The representative lung sections obtained 48 h after intranasal challenge and stained with H&E. (B) The infiltrating inflammation cells were quantified under 400-fold view. The results are expressed as mean ± SEM (n = 5–6 per group) of the cell number per square millimeter of lung tissues. The statistical significance of differences among groups was assessed by the Dunnett’s test. * denoted *p*<0.05.

We further examined the effect of oral feeding of the vaccine on allergic lung inflammation by H&E staining in mice. Histopathologic features of the mouse lungs from each group are shown in **Fig 7A**. There was obvious airway epithelium hyperemia and edema, as well as inflammatory infiltration into the lungs of the sham and vehicle groups of mice. Only the hypoallergen Per a 2–372 group showed a significant reduction in total inflammatory cell infiltration, especially eosinophils (**Fig 7B**). The results revealed that oral feeding of plant extract with Per a 2–372 hypoallergen-encoding plant vaccine suppressed the development of airway inflammation.

## Discussion

In the present study, we constructed a TuMV infectious plant virus to express American cockroach major allergen Per a 2 in Chinese cabbage using a full-clone major allergen Per a 2–996 (containing all IgE binding epitopes) and a partial clone allergen Per a 2–372 that containing only one IgE-binding epitope from our previous study (31). In this murine allergic asthma model, though pre-treated with allergen- or hypoallergen-containing cabbage blunt the development of allergen-specific IgE and IL-4 mRNA expression after challenge, only the hypoallergen, Per a 2–372 had preventive effects on airway hyperresponsiveness and inflammation.

In addition to the effect on the development of allergen-specific IgE after challenge at week 9, only hypoallergen Per a 2–372 significantly down regulate the mRNA expression of the two major Th2 cytokines IL-4 and IL-13 while comparing with the sham group and vector alone group. It seems that the cockroach allergen-encoding cabbage up-regulate the mRNA expression of proinflammatory cytokines from the innate immune cells IL-6 and TNF-alpha in a reverse pattern of the Th2 cytokines (Per a 2–232> Per a 2–996). Though IL-6 and TNF-alpha have been known to play important roles in asthma [32], the paradoxical change of these proinflammatory cytokines did not seem to affect the inflammatory process in the airways according to the histopathology findings in the lung as shown on Fig 7 and airway hyper-responsiveness as shown at Fig 5. Human clinical studies using anti-TNF alpha inhibitors did not show clinical efficacy. There were even reports regarding etanercept and adalimumab -induced asthma for patients using these TNF-alpha inhibitors for other indications [33–37].

If we look at the human clinical studies for emerging monoclonal antibodies in treating asthma for the past 20 years, studies targeting IgE, IL-2, IL4R-alpha, IL-5, and IL-13 showed some efficacy, however those targeting TNF-alpha, TSLP and IL-9 did not show convincing effectiveness [38] and no asthma clinical trials targeting IL-6 have been conducted to the best of our knowledge. It is likely that TNF-alpha and IL-6 may not be the culprits of allergic airway inflammation and may be merely one of the biomarkers participating the inflammatory cascade.

An interesting finding in this study was that plant encoding hypoallergen Per a 2–372 consistently gave better effects in terms of inhibition of development of allergen-specific IgE production, airway hyperresponsiveness, type 2 cytokine production, as well as inhibition of lung inflammation, in comparison with the effects of plant encoding full-length allergen Per a 2–996. Whether plant vaccine encoding IgE-binding epitopes only is more immunogenic, and therefore more effective, than a vaccine encoding full-length allergen, requires further investigation.

Several sublingual and oral allergen-specific immunotherapy vaccines have been developed for treating allergic airway diseases in recent years [39–41]. However, the amount of antigen administered to achieve oral tolerance to allergens is large and the cost remains a major consideration. Plant-made edible vaccination is a promising strategy which offers the advantages of low cost and the potential for mass production [26, 42, 43]. However, further study is required to determine whether this plant vaccine is able to treat pre-existing allergic diseases with the same efficacy as conventional allergen-specific vaccines or sublingual allergen vaccines. Furthermore, it may not be possible to generalize the results of this study to other allergens and other edible plants.

In summary, this is the first report to describe the successful use of a TuMV viral vector-expressed cockroach allergen Per a 2 given as an oral vaccine to prevent the development of allergic asthma and allergic inflammation later in life following exposure to cockroach allergen in a mouse model.

## Supporting information

S1 Fig Cytokine levels in rPer a 2-stimulated splenocyte culture supernatants by ELISA. Cytokine protein levels of splenocytes stimulated with rPer a 2 for 5 days. (A) IL-13, (B) IFN-γ, (C) IL-10. Data are expressed as means±SEM (N = 5–6 per group). The statistical significance of differences among groups was assessed by the Dunnett’s test. * denoted *p*<0.05.
(TIF)
