# Supplementary figures and images for: Oral edible plant vaccine containing hypoallergen of American cockroach major allergen Per a 2 prevents roach-allergic asthma in a murine model
Source: PLoS One. 2018 Jul 30;13(7):e0201281. doi: 10.1371/journal.pone.0201281 (PMC6066233; doi:10.1371/journal.pone.0201281)

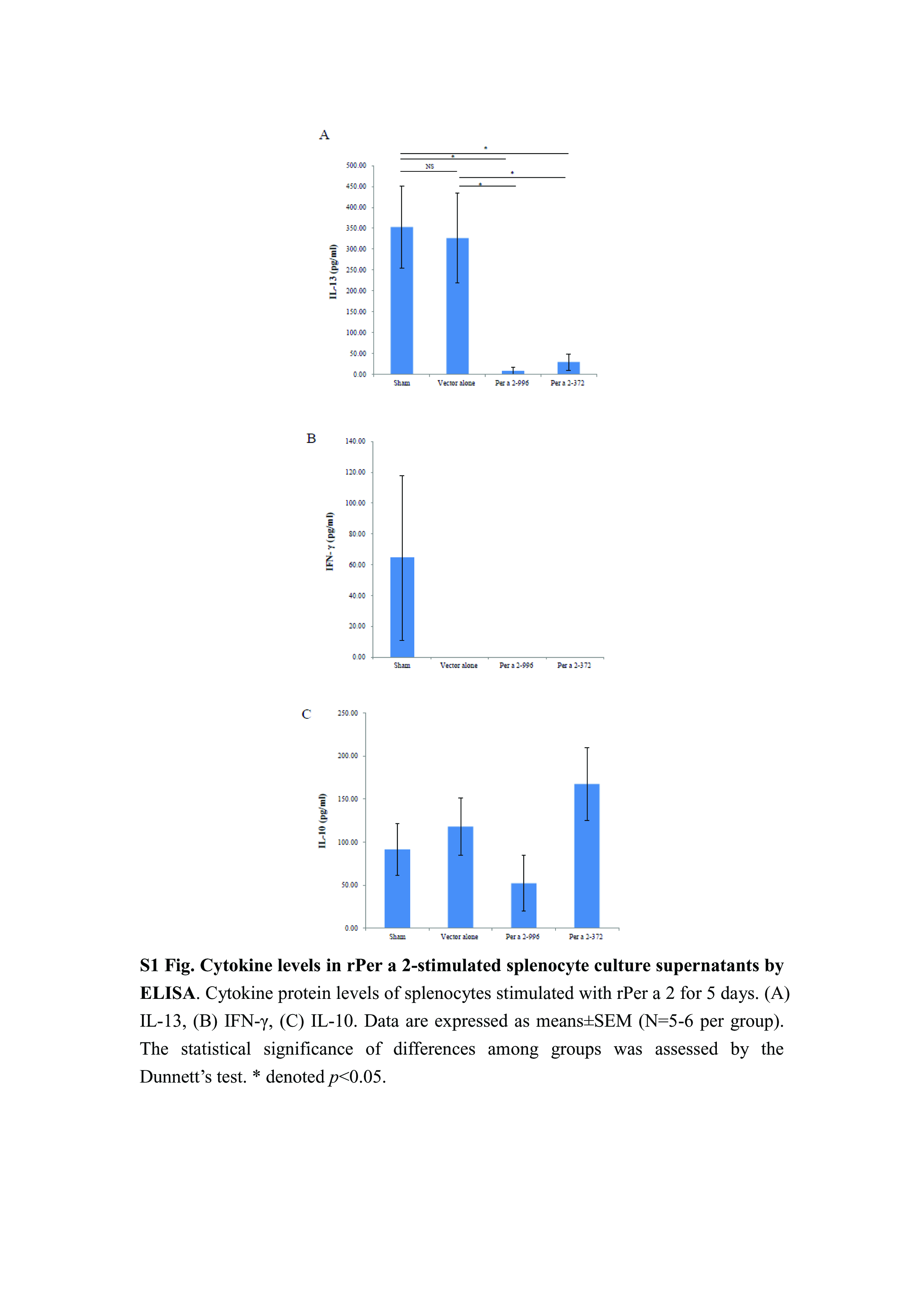

Supplement: S1 Fig — Cytokine protein levels of splenocytes stimulated with rPer a 2 for 5 days. (A) IL-13, (B) IFN-γ, (C) IL-10. Data are expressed as means±SEM (N = 5–6 per group). The statistical significance of differences among groups was assessed by the Dunnett’s test. * denoted p<0.05. (TIF) [file pone.0201281.s001.tif]
